# Supplementary figures and images for: The orphan drug dichloroacetate reduces amyloid beta-peptide production whilst promoting non-amyloidogenic proteolysis of the amyloid precursor protein
Source: PLoS One. 2022 Jan 13;17(1):e0255715. doi: 10.1371/journal.pone.0255715 (PMC8757967; doi:10.1371/journal.pone.0255715)

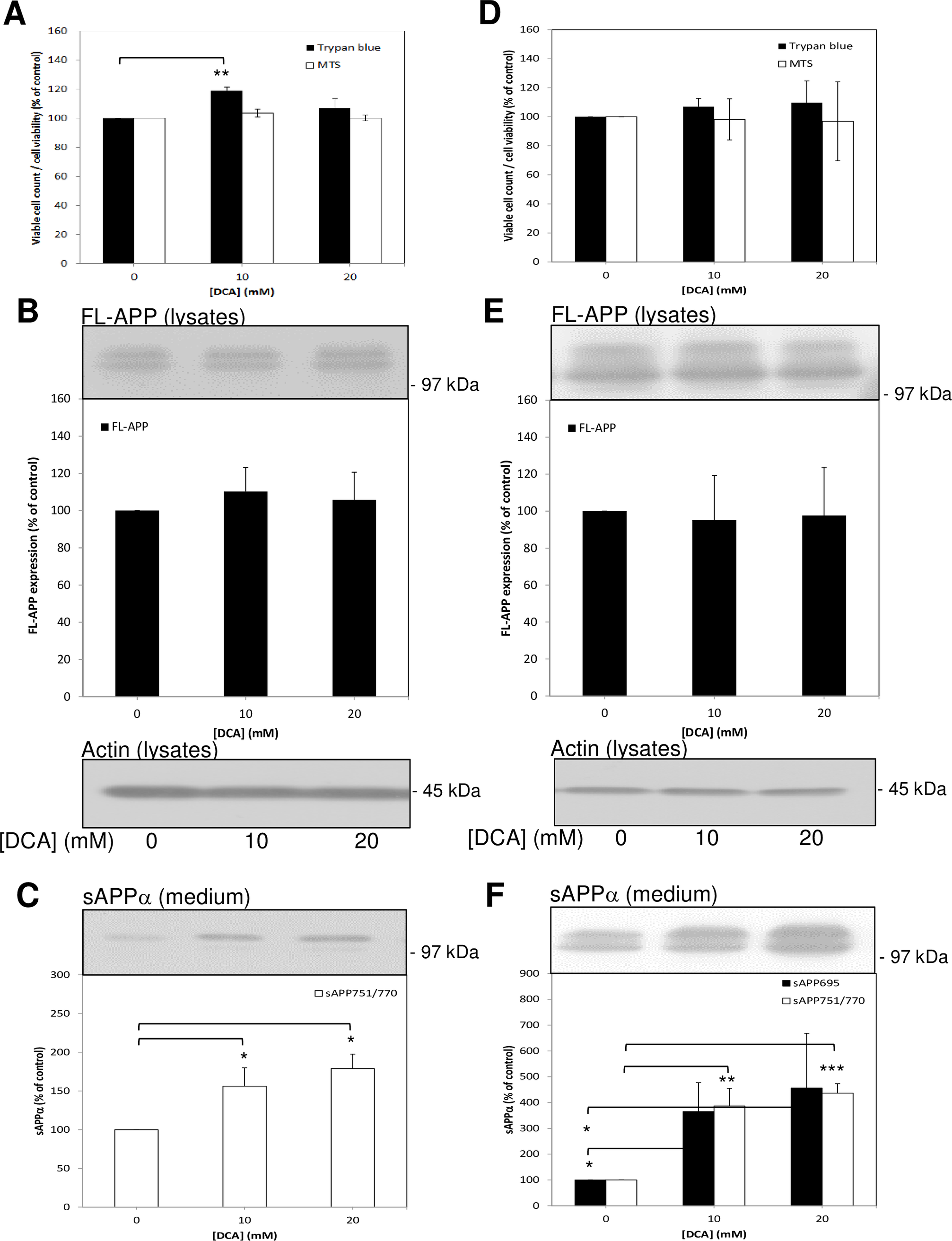

Supplement: S1 Fig — Cells were grown to confluence before replacing the growth medium with UltraMEM containing the indicated DCA concentrations and culturing for an additional 24 h. Viability assays were then performed or cell lysate and conditioned medium samples were prepared as described in the Materials and methods section. Equal amounts of protein (lysates) or equal volumes (medium) from samples were subjected to SDS-PAGE and immunoblotting (Materials and methods). (A) and (D) Trypan blue and MTS cell viability assays. (B and E) Cell lysates were immunoblotted with anti-APP C-terminal antibody and re-probed with anti-actin antibody. Full-length APP (FL-APP) was quantified from multiple immunoblots and the results expressed relative to control values. (C and F) Conditioned medium samples were immunoblotted with anti-APP 6E10 antibody to detect sAPPα. Multiple immunoblots were quantified and results were expressed relative to control values. All results are means ± S.D. (n = 3). * = significant at p < 0.05; ** = significant at p < 0.01; *** = significant at p < 0.005. (TIF) [file pone.0255715.s001.tif]

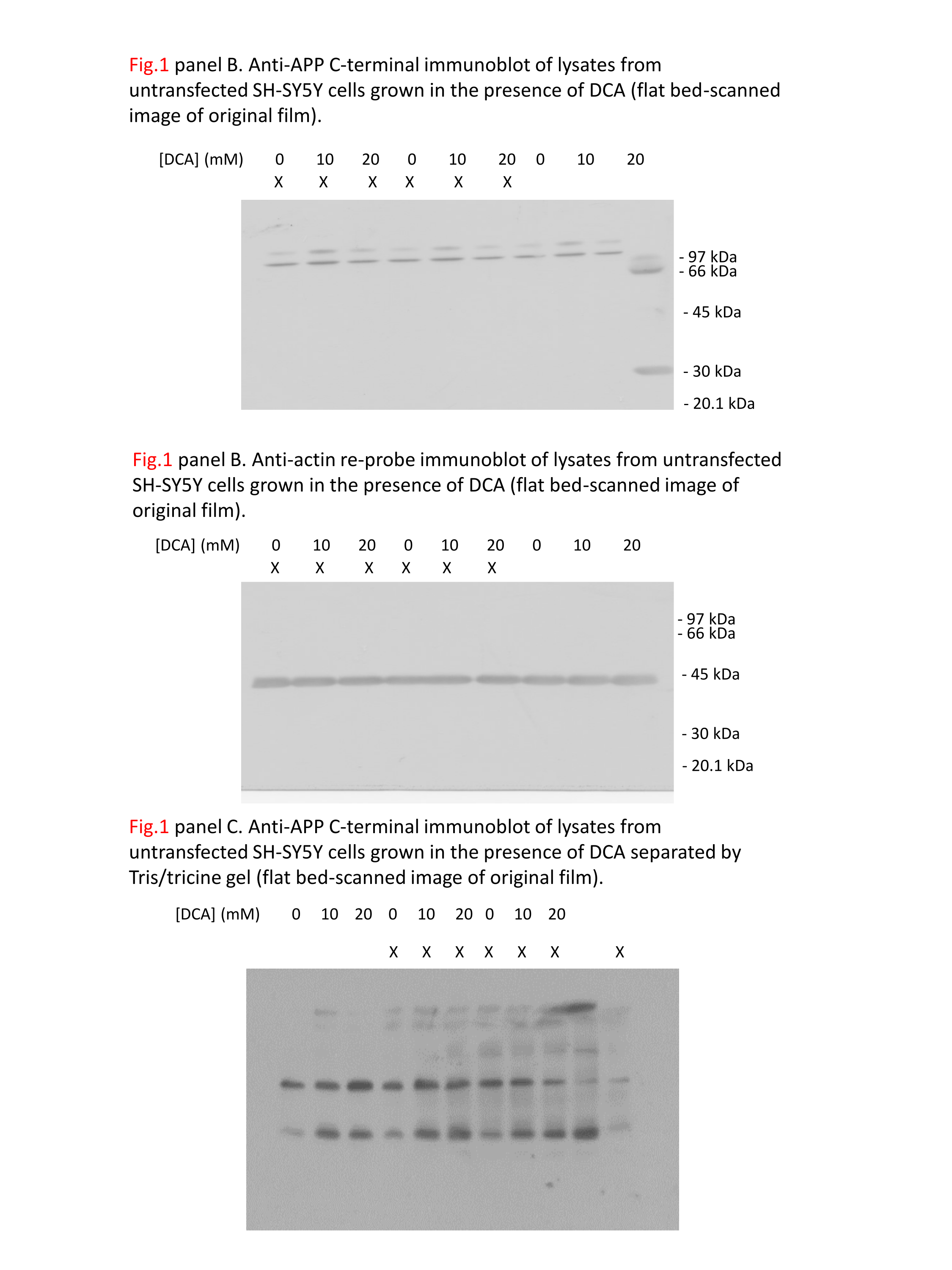

Supplement: S1 Raw images — Whole blot images are shown from which the lanes marked ‘X’ are excluded from cropped images shown in figures. (TIFF) [file pone.0255715.s002.tiff]
