# Supplementary material for: The orphan drug dichloroacetate reduces amyloid beta-peptide production whilst promoting non-amyloidogenic proteolysis of the amyloid precursor protein
Source: PLoS One. 2022 Jan 13;17(1):e0255715. doi: 10.1371/journal.pone.0255715 (PMC8757967; doi:10.1371/journal.pone.0255715)
Supplement: S1 Dataset — Means and standard deviations for individual figure panels along with the statistical testing method and levels of significance. (DOCX) [file pone.0255715.s003.docx]

Where indicated, individual control values are standardized to 100% with treatment repetitions expressed relative to these.

| **Fig. number** | **Mean** | **S.D.** | **Statistical method used** | **P value** | **Number of samples** |
| --- | --- | --- | --- | --- | --- |
| **Fig. 1A** |  |  | one-way analysis of variance (ANOVA) with Tukey’s post hoc tests | ***P ˂0.005 |  |
| Untreated controls  (standardized) | 100 | 0 |  |  | 3 |
| 10 mM DCA (Trypan blue) | 74.98 | 1.37 |  |  | 3 |
| 20 mM DCA (Trypan blue) | 55.07 | 7.89 |  |  | 3 |
| 10 mM DCA (MTS) | 98.28 | 16.49 |  |  | 3 |
| 20 mM DCA (MTS) | 60.00 | 6.02 |  |  | 3 |
|  |  |  |  |  |  |
| **Fig. 1B** |  |  | one-way analysis of variance (ANOVA) with Tukey’s post hoc tests | no significance |  |
| Untreated controls (standardized) | 100 | 0 |  |  | 3 |
| 10 mM DCA | 96.00 | 2.62 |  |  | 3 |
| 20 mM DCA | 109.61 | 7.89 |  |  | 3 |
|  |  |  |  |  |  |
| **Fig. 1C** |  |  | one-way analysis of variance (ANOVA) with Tukey’s post hoc tests | ***P ˂0.005 |  |
| Untreated controls (standardized) | 100 | 0 |  |  | 3 |
| 10 mM DCA | 243.44 | 17.65 |  |  | 3 |
| 20 mM DCA | 213.68 | 12.13 |  |  | 3 |
|  |  |  |  |  |  |
| **Fig. 1D** |  |  | one-way analysis of variance (ANOVA) with Tukey’s post hoc tests | *P ˂0.05 |  |
| Untreated controls (standardized) | 100 | 0 |  |  | 3 |
| 10 mM DCA (sAPP695) | 159.17 | 27.12 |  |  | 3 |
| 20 mM DCA (sAPP695) | 107.82 | 18.92 |  |  | 3 |
| 10 mM DCA (sAPP751/770) | 142.13 | 23.98 |  |  | 3 |
| 20 mM DCA (sAPP751/770) | 121.87 | 19.16 |  |  | 3 |
|  |  |  |  |  |  |
| **Fig. 1E** |  |  | one-way analysis of variance (ANOVA) with Tukey’s post hoc tests | *P ˂0.05 |  |
| Untreated controls (standardized) | 100 | 0 |  |  |  |
| 10 mM DCA (sAPP695) | 225.62 | 36.16 |  |  | 3 |
| 20 mM DCA (sAPP695) | 195.79 | 34.36 |  |  | 3 |
| 10 mM DCA (sAPP751/770) | 189.56 | 31.98 |  |  | 3 |
| 20 mM DCA (sAPP751/770) | 221.30 | 34.79 |  |  | 3 |
|  |  |  |  |  |  |
| **Fig. 2A** |  |  | one-way analysis of variance (ANOVA) with Tukey’s post hoc tests | **P ˂0.01 |  |
| Untreated controls (standardized) | 100 | 0 |  |  | 3 |
| 10 mM DCA (sAPP695) | 0 | 0 |  |  | 3 |
| 20 mM DCA (sAPP695) | 0 | 0 |  |  | 3 |
| 10 mM DCA (sAPP751/770) | 38.12 | 12.16 |  |  | 3 |
| 20 mM DCA (sAPP751/770) | 15.20 | 15.67 |  |  | 3 |
|  |  |  |  |  |  |
| **Fig. 2B** |  |  | one-way analysis of variance (ANOVA) with Tukey’s post hoc tests | **P ˂0.01 |  |
| Untreated controls (standardized) | 100 | 0 |  |  | 3 |
| 10 mM DCA (sAPP695) | 0 | 0 |  |  | 3 |
| 20 mM DCA (sAPP695) | 0 | 0 |  |  | 3 |
| 10 mM DCA (sAPP751/770) | 50.84 | 16.22 |  |  | 3 |
| 20 mM DCA (sAPP751/770) | 27.56 | 28.62 |  |  | 3 |
|  |  |  |  |  |  |
| **Fig. 2C** |  |  | one-way analysis of variance (ANOVA) with Tukey’s post hoc tests | **P ˂0.01  ****P ˂0.001  *****P ˂0.0005 |  |
| (Values in pg/ml) |  |  |  |  |  |
| Untreated control (Aβ1-40) | 59.88 | 6.20 |  |  | 3 |
| 10 mM DCA (Aβ1-40) | 18.29 | 2.78 |  |  | 3 |
| 20 mM DCA (Aβ1-40) | 3.16 | 1.35 |  |  | 3 |
| Untreated control (Aβ1-42) | 5.04 | 0.68 |  |  | 3 |
| 10 mM DCA (Aβ1-42) | 2.07 | 0.63 |  |  | 3 |
| 20 mM DCA (Aβ1-42) | 0.54 | 0.04 |  |  | 3 |
|  |  |  |  |  |  |
| **Fig. 2D** |  |  | one-way analysis of variance (ANOVA) with Tukey’s post hoc tests | **P ˂0.01  ***P ˂0.005  *****P ˂0.0005 |  |
| (Values in pg/ml) |  |  |  |  |  |
| Untreated control (Aβ1-40) | 59.88 | 6.20 |  |  | 3 |
| 10 mM DCA (Aβ1-40) | 24.19 | 3.61 |  |  | 3 |
| 20 mM DCA (Aβ1-40) | 5.75 | 2.45 |  |  | 3 |
| Untreated control (Aβ1-42) | 5.04 | 0.68 |  |  | 3 |
| 10 mM DCA (Aβ1-42) | 2.76 | 0.84 |  |  | 3 |
| 20 mM DCA (Aβ1-42) | 0.99 | 0.07 |  |  | 3 |
|  |  |  |  |  |  |
| **Fig. 3A** |  |  | one-way analysis of variance (ANOVA) with Tukey’s post hoc tests | *P ˂0.05 |  |
| Untreated controls  (standardized) | 100 | 0 |  |  | 3 |
| 10 mM DCA (Trypan blue) | 93.78 | 1.44 |  |  | 3 |
| 20 mM DCA (Trypan blue) | 108.85 | 4.18 |  |  | 3 |
| 10 mM DCA (MTS) | 104.39 | 2.81 |  |  | 3 |
| 20 mM DCA (MTS) | 93.52 | 7.58 |  |  | 3 |
|  |  |  |  |  |  |
| **Fig. 3B** |  |  | one-way analysis of variance (ANOVA) with Tukey’s post hoc tests | **P ˂0.01 |  |
| Untreated controls (standardized) | 100 | 0 |  |  | 3 |
| 10 mM DCA | 127.00 | 5.83 |  |  | 3 |
| 20 mM DCA | 129.00 | 33.62 |  |  | 3 |
|  |  |  |  |  |  |
| **Fig. 3C** |  |  | one-way analysis of variance (ANOVA) with Tukey’s post hoc tests | ***P ˂0.005  *****P ˂0.0005 |  |
| Untreated controls (standardized) | 100 | 0 |  |  | 3 |
| 10 mM DCA | 596.45 | 59.02 |  |  | 3 |
| 20 mM DCA | 623.10 | 5.62 |  |  | 3 |
|  |  |  |  |  |  |
| **Fig. 3D** |  |  | one-way analysis of variance (ANOVA) with Tukey’s post hoc tests | **P ˂0.01 |  |
| Untreated controls (standardized) | 100 | 0 |  |  | 3 |
| 10 mM DCA | 20.13 | 13.17 |  |  | 3 |
| 20 mM DCA | 0 | 0 |  |  | 3 |
|  |  |  |  |  |  |
| **Fig. 3E** |  |  | one-way analysis of variance (ANOVA) with Tukey’s post hoc tests | **P ˂0.01  ***P ˂0.005  ****P ˂0.001 |  |
| (Values in pg/ml) |  |  |  |  |  |
| Untreated control (Aβ1-40) | 76.74 | 7.86 |  |  | 3 |
| 10 mM DCA (Aβ1-40) | 50.87 | 0.63 |  |  | 3 |
| 20 mM DCA (Aβ1-40) | 30.09 | 1.98 |  |  | 3 |
| Untreated control (Aβ1-42) | 6.35 | 0.16 |  |  | 3 |
| 10 mM DCA (Aβ1-42) | 4.77 | 0.14 |  |  | 3 |
| 20 mM DCA (Aβ1-42) | 3.04 | 0.10 |  |  | 3 |
|  |  |  |  |  |  |
| **Fig. 4A** |  |  | one-way analysis of variance (ANOVA) with Tukey’s post hoc tests | *P ˂0.05 |  |
| Untreated controls  (standardized) | 100 | 0 |  |  | 3 |
| 10 mM DCA (Trypan blue) | 97.55 | 3.16 |  |  | 3 |
| 20 mM DCA (Trypan blue) | 99.39 | 3.87 |  |  | 3 |
| 10 mM DCA (MTS) | 93.85 | 3.21 |  |  | 3 |
| 20 mM DCA (MTS) | 85.62 | 8.54 |  |  | 3 |
|  |  |  |  |  |  |
| **Fig. 4B** |  |  | one-way analysis of variance (ANOVA) with Tukey’s post hoc tests | No significance |  |
| Untreated controls (standardized) | 100 | 0 |  |  | 3 |
| 10 mM DCA | 111.89 | 8.17 |  |  | 3 |
| 20 mM DCA | 116.16 | 18.76 |  |  | 3 |
|  |  |  |  |  |  |
| **Fig. 4C** |  |  | one-way analysis of variance (ANOVA) with Tukey’s post hoc tests | **P ˂0.01 |  |
| Untreated controls (standardized) | 100 | 0 |  |  | 3 |
| 10 mM DCA | 182.79 | 15.99 |  |  | 3 |
| 20 mM DCA | 225.99 | 33.63 |  |  | 3 |
|  |  |  |  |  |  |
| **Fig. 4D** |  |  | one-way analysis of variance (ANOVA) with Tukey’s post hoc tests | No significance |  |
| Untreated controls (standardized) | 100 | 0 |  |  | 3 |
| 10 mM DCA | 98.78 | 8.11 |  |  | 3 |
| 20 mM DCA | 80.16 | 18.91 |  |  | 3 |
|  |  |  |  |  |  |
| **Fig. 4E** |  |  | one-way analysis of variance (ANOVA) with Tukey’s post hoc tests | *P ˂0.05  **P ˂0.01 |  |
| (Values in pg/ml) |  |  |  |  |  |
| Untreated control (Aβ1-40) | 431.37 | 47.09 |  |  | 3 |
| 10 mM DCA (Aβ1-40) | 390.16 | 51.36 |  |  | 3 |
| 20 mM DCA (Aβ1-40) | 289.14 | 25.69 |  |  | 3 |
| Untreated control (Aβ1-42) | 37.74 | 2.15 |  |  | 3 |
| 10 mM DCA (Aβ1-42) | 33.95 | 2.87 |  |  | 3 |
| 20 mM DCA (Aβ1-42) | 28.30 | 1.69 |  |  | 3 |
|  |  |  |  |  |  |
| **Fig. 5A** |  |  | one-way analysis of variance (ANOVA) with Tukey’s post hoc tests | **P ˂0.01 |  |
| Untreated controls  (standardized) | 100 | 0 |  |  | 3 |
| 10 mM DCA (Trypan blue) | 94.67 | 5.94 |  |  | 3 |
| 20 mM DCA (Trypan blue) | 94.23 | 7.97 |  |  | 3 |
| 10 mM DCA (MTS) | 91.91 | 3.76 |  |  | 3 |
| 20 mM DCA (MTS) | 70.18 | 4.02 |  |  | 3 |
|  |  |  |  |  |  |
| **Fig. 5B** |  |  | one-way analysis of variance (ANOVA) with Tukey’s post hoc tests | *P ˂0.05 |  |
| Untreated controls (standardized) | 100 | 0 |  |  | 3 |
| 10 mM DCA | 117.31 | 12.98 |  |  | 3 |
| 20 mM DCA | 162.31 | 22.78 |  |  | 3 |
|  |  |  |  |  |  |
| **Fig. 5C** |  |  | one-way analysis of variance (ANOVA) with Tukey’s post hoc tests | No significance |  |
| Untreated controls (standardized) | 100 | 0 |  |  | 3 |
| 10 mM DCA | 105.23 | 9.54 |  |  | 3 |
| 20 mM DCA | 108.89 | 12.26 |  |  | 3 |
|  |  |  |  |  |  |
| **Fig. 5D** |  |  | one-way analysis of variance (ANOVA) with Tukey’s post hoc tests | **P ˂0.01  ***P ˂0.005 |  |
| Untreated controls (standardized) | 100 | 0 |  |  | 3 |
| 10 mM DCA (sAPP695) | 556.77 | 21.89 |  |  | 3 |
| 20 mM DCA (sAPP695) | 446.19 | 67.13 |  |  | 3 |
| 10 mM DCA (sAPP751/770) | 532.15 | 27.83 |  |  | 3 |
| 20 mM DCA (sAPP751/770) | 516.82 | 33.61 |  |  | 3 |
|  |  |  |  |  |  |
| **Fig. 5E** |  |  | one-way analysis of variance (ANOVA) with Tukey’s post hoc tests | No significance |  |
| Untreated controls (standardized) | 100 | 0 |  |  | 3 |
| 10 mM DCA (sAPP695) | 106.66 | 19.96 |  |  | 3 |
| 20 mM DCA (sAPP695) | 111.12 | 16.23 |  |  | 3 |
| 10 mM DCA (sAPP751/770) | 94.67 | 27.12 |  |  | 3 |
| 20 mM DCA (sAPP751/770) | 98.22 | 16.93 |  |  | 3 |
|  |  |  |  |  |  |
| **Fig. 5F** |  |  | one-way analysis of variance (ANOVA) with Tukey’s post hoc tests | *P ˂0.05 |  |
| (Values in pg/ml) |  |  |  |  |  |
| Untreated control (Aβ1-40) | 285.00 | 31.68 |  |  | 3 |
| 10 mM DCA (Aβ1-40) | 286.52 | 37.06 |  |  | 3 |
| 20 mM DCA (Aβ1-40) | 189.70 | 28.80 |  |  | 3 |
| Untreated control (Aβ1-42) | 25.62 | 2.73 |  |  | 3 |
| 10 mM DCA (Aβ1-42) | 25.80 | 5.50 |  |  | 3 |
| 20 mM DCA (Aβ1-42) | 17.65 | 1.82 |  |  | 3 |
|  |  |  |  |  |  |
| **Fig. 6B** |  |  | one-way analysis of variance (ANOVA) with Tukey’s post hoc tests | No significance |  |
| Values (Relative normalized expression) |  |  |  |  |  |
| ADAM10 (untreated) | 1.10 | 0.20 |  |  | 3 |
| ADAM10 (10 mM DCA) | 0.89 | 0.17 |  |  | 3 |
| ADAM10 (20 mM DCA) | 0.82 | 0.15 |  |  | 3 |
| BACE1 (untreated) | 1.13 | 0.16 |  |  | 3 |
| BACE1 (10 mM DCA) | 0.93 | 0.18 |  |  | 3 |
| BACE1 (20 mM DCA) | 0.97 | 0.15 |  |  | 3 |
| PS-1 (untreated) | 1.16 | 0.10 |  |  | 3 |
| PS-1 (10 mM DCA) | 0.99 | 0.03 |  |  | 3 |
| PS-1 (20 mM DCA) | 1.01 | 0.23 |  |  | 3 |
|  |  |  |  |  |  |
| **Fig. 6C** |  |  | one-way analysis of variance (ANOVA) with Tukey’s post hoc tests | No significance |  |
| Untreated controls (standardized) | 100 | 0 |  |  | 6 |
| ADAM10 (10 mM DCA) | 87.92 | 13.27 |  |  | 6 |
| ADAM10 (20 mM DCA) | 91.65 | 16.93 |  |  | 6 |
| BACE1 (10 mM DCA) | 71.83 | 14.50 |  |  | 6 |
| BACE1 (20 mM DCA) | 70.99 | 19.58 |  |  | 6 |
|  |  |  |  |  |  |
| **Fig. 7A** |  |  | one-way analysis of variance (ANOVA) with Tukey’s post hoc tests | *P ˂0.05 |  |
| Untreated controls (standardized) | 100 | 0 |  |  | 3 |
| 10 mM DCA (FL-Jagged1) | 112.78 | 24.77 |  |  | 3 |
| 20 mM DCA (FL-Jagged1) | 85.12 | 55.31 |  |  | 3 |
| 10 mM DCA (Jagged1-CTF) | 148.96 | 26.21 |  |  | 3 |
| 20 mM DCA (Jagged1-CTF) | 198.15 | 48.12 |  |  | 3 |
|  |  |  |  |  |  |
| **Fig. 7B** |  |  | one-way analysis of variance (ANOVA) with Tukey’s post hoc tests | **P ˂0.01 |  |
| Untreated controls (standardized) | 100 | 0 |  |  | 3 |
| 10 mM DCA | 221.50 | 12.56 |  |  | 3 |
| 20 mM DCA | 245.10 | 26.66 |  |  | 3 |
|  |  |  |  |  |  |
| **Fig. S1 A** |  |  | one-way analysis of variance (ANOVA) with Tukey’s post hoc tests | **P ˂0.01 |  |
| Untreated controls  (standardized) | 100 | 0 |  |  | 3 |
| 10 mM DCA (Trypan blue) | 119.18 | 2.20 |  |  | 3 |
| 20 mM DCA (Trypan blue) | 106.97 | 7.44 |  |  | 3 |
| 10 mM DCA (MTS) | 103.71 | 2.54 |  |  | 3 |
| 20 mM DCA (MTS) | 100.26 | 2.00 |  |  | 3 |
|  |  |  |  |  |  |
| **Fig. S1 B** |  |  | one-way analysis of variance (ANOVA) with Tukey’s post hoc tests | No significance |  |
| Untreated controls (standardized) | 100 | 0 |  |  | 3 |
| 10 mM DCA | 110.26 | 12.89 |  |  | 3 |
| 20 mM DCA | 105.78 | 14.86 |  |  | 3 |
|  |  |  |  |  |  |
| **Fig. S1 C** |  |  | one-way analysis of variance (ANOVA) with Tukey’s post hoc tests | *P ˂0.05 |  |
| Untreated controls (standardized) | 100 | 0 |  |  | 3 |
| 10 mM DCA | 156.12 | 23.87 |  |  | 3 |
| 20 mM DCA | 178.92 | 18.67 |  |  | 3 |
|  |  |  |  |  |  |
| **Fig. S1 D** |  |  | one-way analysis of variance (ANOVA) with Tukey’s post hoc tests | No significance |  |
| Untreated controls  (standardized) | 100 | 0 |  |  | 3 |
| 10 mM DCA (Trypan blue) | 106.91 | 5.78 |  |  | 3 |
| 20 mM DCA (Trypan blue) | 109.69 | 15.05 |  |  | 3 |
| 10 mM DCA (MTS) | 98.19 | 14.18 |  |  | 3 |
| 20 mM DCA (MTS) | 96.89 | 27.21 |  |  | 3 |
|  |  |  |  |  |  |
| **Fig. S1 E** |  |  | one-way analysis of variance (ANOVA) with Tukey’s post hoc tests | No significance |  |
| Untreated controls (standardized) | 100 | 0 |  |  | 3 |
| 10 mM DCA | 95.16 | 24.12 |  |  | 3 |
| 20 mM DCA | 97.63 | 26.12 |  |  | 3 |
|  |  |  |  |  |  |
| **Fig. S1 F** |  |  |  |  |  |
| Untreated controls (standardized) | 100 | 0 | one-way analysis of variance (ANOVA) with Tukey’s post hoc tests | *P ˂0.05  **P ˂0.01  ***P ˂0.005 | 3 |
| 10 mM DCA (sAPP695) | 364.89 | 112.12 |  |  | 3 |
| 20 mM DCA (sAPP695) | 456.12 | 212.12 |  |  | 3 |
| 10 mM DCA (sAPP751/770) | 386.72 | 68.31 |  |  | 3 |
| 20 mM DCA (sAPP751/770) | 436.31 | 36.71 |  |  | 3 |
